# Fibrotic–Angiogenic Signaling Networks in Oral Submucous Fibrosis: Pathobiology and Therapeutic Targeting

**DOI:** 10.3390/ijms27167204

**Published:** 2026-08-12

**Authors:** Samar Kamran, Nabeel Reza, Zurairah Berahim, Saeed Ur Rahman, Johari Yap Abdullah

**Affiliations:** 1 School of Dental Sciences, Health Campus, Universiti Sains Malaysia, Kubang Kerian 16150, Kelantan, Malaysia; samar.kamran@student.usm.my (S.K.); nabeelreza@student.usm.my (N.R.); 2 Oral Biology, Institute of Basic Medical Sciences, Khyber Medical University, Peshawar 25000, Pakistan; saeed.ibms@kmu.edu.pk; 3 Dental Research Unit, Center for Transdisciplinary Research (CFTR), Saveetha Institute of Medical and Technical Sciences, Saveetha Dental College, Saveetha University, Chennai 602105, India

**Keywords:** oral submucous fibrosis, *TGF-β*, *CTGF*, and *COL1A1* signaling, fibrosis–angiogenesis interaction, epithelial dysregulation, malignant transformation, precision medicine

## Abstract

Oral submucous fibrosis (OSMF) is a chronic, progressive fibrotic disorder with significant malignant potential, and limited effective treatments due to an incomplete understanding of its underlying molecular mechanisms. This narrative review synthesizes current evidence on the key molecular networks involved in OSMF pathogenesis, highlighting the central role of the *TGF-β1*/*Smad*, *CTGF*, and *COL1A1* pathways in maintaining fibroblast activation, supporting myofibroblast activity, and promoting pathological collagen accumulation. It also integrates recent findings on how profibrotic signaling interacts with important angiogenic and epithelial growth factors such as *VEGF*, *FGF*, and *EGF*, creating a stage-dependent fibrotic microenvironment. In early OSMF, increased angiogenic signaling, especially through the *VEGF*/PI3K–Akt pathway, helps maintain blood supply. However, as the disease progresses, *TGF-β*-induced pathways become dominant, leading to vascular rarefaction, tissue hypoxia, epithelial instability and worsening fibrosis that increases the risk of malignant transformation. By critically appraising molecular, cellular, and translational studies, this review identifies key pathways linking fibrosis, angiogenesis, and epithelial changes in OSMF. It suggests that stage-specific and combined targeting of *TGF-β1*, *CTGF*, *COL1A1*, along with angiogenic and epithelial pathways, may help reverse fibrosis, restore normal tissue perfusion, reduce cancer risk, and support the development of better therapies and biomarkers beyond symptom relief.

## 1. Introduction

Fibrosis is a dysregulated wound healing process characterized by persistent fibroblast activation leading to excessive extracellular matrix (ECM) deposition and progressive disruption of normal tissue architecture [1,2,3,4,5]. In the oral cavity, this process is seen in oral submucous fibrosis (OSMF), a chronic and debilitating condition with a global prevalence of approximately 4.96% [6,7]. It is more common in South and Southeast Asia and mainly affects young adults [8,9,10,11,12,13]. It is strongly linked to areca nut chewing and has a known risk of progressing to oral squamous cell carcinoma (OSCC), with a malignant transformation rate of around 5 to 13% [9,14,15,16,17,18,19]. The progression of the disease occurs through some distinct stages starting from burning sensation and vesiculation to palpable fibrous bands, mucosal rigidity, and ultimately advanced fibrosis with pronounced trismus and severe oral functional impairment [20,21,22]. Although OSMF has been extensively studied, current therapeutic approaches remain largely symptomatic, reflecting an incomplete understanding of the underlying biological mechanisms that sustain fibrosis and cause disease progression.

At the molecular level, OSMF is marked by sustained activation of fibroblasts and accumulation of myofibroblasts, resulting in excessive collagen type I (*COL1A1*) deposition and impaired matrix turnover [23,24,25,26,27,28,29,30]. Transforming growth factor-*β1* (*TGF-β1*) is widely regarded as the central regulator of this fibrotic response, acting through Smad-dependent and non-canonical signaling pathways to induce connective tissue growth factor (*CTGF*), promote fibroblast to myofibroblast transition, and reinforce extracellular matrix remodeling and consequent *COL1A1* upregulation [31,32,33,34,35,36,37,38,39,40]. Importantly, fibrogenesis in OSMF occurs within a dynamically evolving microenvironment that undergoes parallel vascular and epithelial changes during disease progression. Angiogenic signaling mediated by vascular endothelial growth factor (*VEGF*), together with fibroblast growth factor (*FGF*) and epidermal growth factor (*EGF*) pathways, contributes to tissue adaptation under hypoxic stress and influences epithelial homeostasis, suggesting that fibrotic, vascular, and epithelial processes are mechanistically interconnected, but their roles are not much investigated in the pathophysiology of fibrogenesis [41,42,43,44].

Although the individual roles of *TGF-β*, *CTGF*, *VEGF*, *FGF*, *EGF* and *COL1A1* have been widely reported, their coordinated and stage-dependent interactions linking fibrosis, angiogenesis, and epithelial dysregulation have not been comprehensively integrated. This indicates an important gap in our understanding of how these interconnected signaling pathways work together to carry out disease progression and increase the risk of malignant transformation. Addressing this gap is crucial for the development of mechanism-based therapeutic strategies that can modify disease progression and outcomes rather than only providing symptomatic relief.

In this review, current evidence on the pathogenesis of OSMF has been integrated to present a clear and unified molecular perspective. Specifically, we

- Delineate the central *TGF-β1*/Smad/*CTGF*/collagen axis that drives fibrotic remodeling;
- Explore the interaction between profibrotic and angiogenic signaling pathways involving *VEGF*, *FGF*, and *EGF*;
- Contextualize these molecular interactions in relation to disease progression, emerging therapeutic targets, and the risk of malignant transformation.

By integrating these pathways within a unified conceptual framework, this review aims to provide a clearer mechanistic understanding of how stromal fibrosis, vascular remodeling, and epithelial alterations converge during OSMF progression and to highlight opportunities for biologically targeted intervention. A summary of the key molecular pathways involved in fibrotic and angiogenic dysregulation in OSMF is presented in Table 1.

Detailed study methods supporting this framework are provided in Table 2.

### 1.1. The Central Role of TGF-β1, CTGF and COL1A1 in OSMF Pathogenesis

#### 1.1.1. *TGF-β1*

*TGF-β1* is well recognized as the main profibrotic mediator in OSMF, acting as a master regulator of fibrotic process. It exhibits consistently elevated expression in diseased tissues relative to the normal oral mucosa, with levels correlating with histopathological severity [24,35,45,53]. Elevated *TGF-β1* expression, together with strong nuclear p-Smad2 immunoreactivity, confirms activation of canonical *TGF-β* signaling in OSMF tissues [36].

Areca nut exposure plays a pivotal role in activating this pathway. In oral epithelial (HaCaT) cells, it induces the SMAD2 pathway and enhances profibrotic gene expression. Upregulation of *TGF-β2* and *THBS1* concurrently by its alkaloid and polyphenolic constituents, promotes activation of latent *TGF-β* that reinforces a key pathogenic mechanism in OSMF progression [37]. Moreover, dysregulation of the *TGF-β*/Smad pathway further intensifies this signaling cascade. Upregulation of microRNAs-424 (miR-424) suppresses *TGIF2*, an endogenous repressor of the *TGF-β* signaling, and thereby enhances *TGF-β* induced myofibroblast activation and extracellular matrix accumulation, resulting in sustained fibrogenic signaling in OSMF [76]. Emerging evidence also highlights the active role of epithelial cells, where arecoline-induced cellular senescence produces a *TGF-β1*-rich senescence-associated secretory phenotype (SASP) that promotes myofibroblast activation and ECM deposition [46].

Consistent with these findings, integrated quantitative polymerase chain reaction (qPCR) and immunohistochemical studies demonstrate significant upregulation of all three TGF-β isoforms (*β1*, *β2*, *β3*) and their receptors (*TGF-βR1* and *TGF-βR2*) in OSMF tissues compared to normal oral mucosa, with *TGF-β1* exhibiting the most pronounced increase [35]. Immunohistochemical studies further reveal that both *TGF-β1* and *TGF-β2* are upregulated in OSMF. Expression of *TGF-β1* is widespread and plays a major role in fibrosis, while *TGF-β2*, mainly present in the submucosa, also contributes and supports this effect [34].

Notably, salivary levels of *TGF-β* isoforms (*TGF-β1*, *TGF-β2*, and *TGF-β3*) are significantly elevated in patients with OSMF and show a stage-dependent increase, highlighting their potential as non-invasive biomarkers. Following therapy, significant reductions in salivary *TGF-β* levels particularly *TGF-β1*, correlates with clinical improvement, further supporting its role as both a key pathogenic fibrotic mediator and a marker of disease severity [79].

#### 1.1.2. *CTGF*: The Critical Downstream Mediator

*CTGF*/*CCN2* is a crucial downstream mediator of *TGF-β1*-mediated fibrogenesis and plays a central role in the pathogenesis of OSMF [83,84,85]. As a matricellular protein, *CTGF* regulates cellular adhesion, migration, proliferation, and ECM synthesis [54,86]. Numerous research studies demonstrated progressive upregulation of *CTGF* in OSMF tissues, with strong correlation of expression levels to the stage and severity of the disease [87,88,89]. Notably, arecoline induces *CTGF*/*CCN2* expression in human buccal mucosal fibroblasts in a dose- and time-dependent manner, identifying it as a key mediator of areca nut-induced fibrogenesis [90].

The association of *TGF-β1* and *CTGF* expression levels has been thoroughly investigated. Experimental evidence indicates that *CTGF* is essential for *TGF-β1*-induced fibroblast-to-myofibroblast differentiation, as its suppression inhibits α-SMA and collagen I expression. In contrast, overexpression of Smad7 inhibits *TGF-β1* signaling by blocking Smad2 phosphorylation. Leading to decreased expression of *CTGF*, alpha-smooth muscle actin (α-SMA), and type I collagen. This highlights *CTGF* as a crucial driver of *TGF-β1*-induced myofibroblast differentiation [48].

*CTGF* expressions are also modulated by upstream regulatory pathways. Glycogen synthase kinase-3 beta (GSK-3β)-dependent signaling is required for *TGF-β1*-induced *CTGF* activation; however, GSK-3β is inhibited by activation of the Wnt/β-catenin pathway, thereby suppressing *CTGF* upregulation [49]. In parallel, phenytoin-induced fibrosis has been shown to induce activation of latent *TGF-β1* via NOX4-derived reactive oxygen species, promoting *CTGF* expression, fibroblast activation, and ECM deposition in human gingival fibroblasts. Disruption of the NOX4–ROS–*TGF-β1*/Smad3 axis effectively suppresses *CTGF* induction, demonstrating its central role in fibrotic signaling [74].

Consistent with these findings, *CTGF* is markedly upregulated in OSMF tissues in conjunction with *TGF-β1* [36,83,85]. An in-depth spatial analysis further reveals minimal expression of *CTGF* in normal oral mucosa with progressive increases in epithelial, stromal, perivascular, and muscular tissues in OSMF, reaching peak levels in OSMF associated well differentiated squamous cell carcinoma (WDSCC). This gradual upregulation suggests that *CTGF* not only promotes fibrosis but may also play a role in malignant transformation [54]. In addition to its role in fibrosis, *CTGF*/*CCN2* contributes to the angiogenic imbalance in OSMF by modulating *VEGF* signaling [54,60,91,92,93].

#### 1.1.3. *COL1A1* and Excessive Collagen Deposition

Type I collagen, primarily encoded by the *COL1A1* gene, is the predominant ECM component found in OSMF [56,94,95]. Its excessive deposition is regarded as a hallmark histopathological feature of the disease and has been closely linked to clinical signs such as mucosal rigidity, restricted mouth opening, and impaired oral function [96,97].

Ultrastructural studies using atomic force microscopy demonstrated a densely packed collagen fibrillar matrix, mainly type 1 collagen, in the subepithelial connective tissue of OSMF, along with disrupted collagen architecture. This remodeling results in a significant increase in oral mucosal stiffness and reduced flexibility, as indicated by elevated Young’s modulus, stiffness, and adhesiveness compared with normal oral mucosa. These nanomechanical changes are aligned with a profibrotic matrix composition characterized by increased collagen I, reduced collagen III and fibronectin, and α-SMA positive myofibroblast accumulation [98].

Progressive collagen I deposition extends from the lamina propria into deeper submucosal layers with advancing disease severity. RNA sequencing and immunofluorescence demonstrated cartilage oligomeric matrix protein (COMP) as a key profibrotic mediator that promotes collagen I synthesis by OSMF fibroblasts, thus further contributing to ECM accumulation [95].

Consistent with this, multiple studies demonstrate stage-dependent upregulation of collagen I expression in OSMF [99,100]. A study demonstrated a gradual, stage-dependent increase in subepithelial collagen deposition from normal oral mucosa through non-dysplastic-to-dysplastic oral submucous fibrosis. These stromal changes were accompanied by a progressive reduction in epithelial E-cadherin expression and a concomitant upregulation of p63, particularly in dysplastic OSMF. These findings indicate a strong correlation between collagen accumulation and epithelial dysregulation, during OSMF progression and dysplastic transformation [101].

At the regulatory level, long non-coding RNAs (lncRNA) contribute to the modulation of collagen expression. Upregulation of lncRNA H19, coupled with downregulation of miR-29b, enhances *COL1A1* expression in OSMF fibroblasts. Knockdown of H19 restores miR-29b levels and correspondingly decreases *COL1A1* expression. Luciferase assays validated that miR-29b exerts direct inhibitory effects on *COL1A1* translation, thereby establishing it as a promising target for antifibrotic intervention [55]. In nutshell, extensive research on the role of *COL1A1* in OSMF provides a basis for follow-up research and potential therapeutic targets [102].

In addition to increased synthesis, impaired collagen degradation significantly contributes to the net accumulation of the ECM. Dysregulation of matrix metalloproteinases (MMPs) and tissue inhibitors of metalloproteinases (TIMPs) results in ECM accumulation and stabilization rather than degradation of collagen, thereby reinforcing fibrotic progression in OMSF [103,104,105].

### 1.2. Angiogenic Dysregulation: The VEGF, FGF, and EGF Paradox

#### 1.2.1. *VEGF*: Biphasic Expression and Vascular Rarefaction

*VEGF* plays a complex and paradoxical role in the pathogenesis of OSMF. Although *VEGF* is predominantly known as a pro-angiogenic factor that facilitates endothelial cell viability, proliferation, and vascular permeability [106], its expression profile in OSMF presents a unique biphasic pattern that is closely associated with the progression of the disease [107,108].

In early-stage OSMF, *VEGF* signaling, in conjunction with phosphoinositide 3-kinase and phosphorylated Akt (PI3K/Akt pathway) is upregulated, likely reflecting a compensatory angiogenic response to hypoxia and metabolic stress induced by areca nut exposure [57]. This is consistent with observations that vascularity in early OSMF may be preserved or even increased as an adaptive response to transient hypoxia [59,109].

However, as fibrosis advances, extensive extracellular matrix accumulation and stromal hyalinization disrupt vascular architecture and impair effective perfusion despite the presence of micro vessels [109]. Consequently, vessel density and effective perfusion decline despite persistent or elevated *VEGF* expression, alongside progressive microvascular dysfunction and increased collagen I deposition, marking a transition from adaptive angiogenesis to functional vascular insufficiency [57,59,110].

Significantly elevated circulating Hypoxia-Inducible Factor-1 Alpha (HIF-1α) and *VEGF* levels in areca nut users with OSMF further support the link between areca nut exposure, hypoxia, and angiogenic activation [42,111].

Despite elevated *VEGF* expression, advanced OSMF is paradoxically characterized by reduced vascularity, a phenomenon described as the angiogenic–fibrotic paradox. Mechanistically, arecoline-induced upregulation of *TGF-β1* promotes thrombospondin-1 (*THBS1*) expression, which inhibits *VEGF*-mediated endothelial sprouting via CD36 signaling, leading to vascular rarefaction despite persistent *VEGF* presence. [60,112]. This indicates that VEGF availability alone is insufficient to sustain angiogenesis in the context of progressive fibrosis and anti-angiogenic signaling [113]. In the absence of dysplastic change, *VEGF* thus exhibits a stage-dependent, dysregulated role in OSMF, supporting early angiogenic responses but becoming functionally ineffective in advanced disease due to dominant fibrotic and anti-angiogenic mediators like XIIIa- and MMP-9-induced matrix remodeling [114].

This general decline, however, is not uniform across all OSMF lesions: in the subset of lesions undergoing dysplastic progression, *VEGF* expression, together with increased Endoglin (cluster of differentiation 105) (CD105)-positive neovascularization, has instead been found to rise, marking a second, malignancy-associated angiogenic phase distinct from the broader fibrotic decline described above. In this specific context, *VEGF* appears to be repurposed away from its adaptive, perfusion-supporting role in early OSMF and toward supporting the neovascularization required for malignant transformation, rather than tracking the general vascular decline [115]. In the more common, non-dysplastic trajectory, this progressive vascular dysfunction has been correlated with increasing fibrosis and is manifested clinically as mucosal blanching, stiffness, and restricted mouth opening [30,109].

Taken together, these findings indicate that OSMF is characterized not by simple, uniform vascular loss, but by maladaptive and context-dependent vascular remodeling: early compensatory angiogenesis is progressively overwhelmed by the mechanical and biochemical constraints of advancing fibrosis in most lesions, while a distinct, focal re-emergence of *VEGF* signaling marks the subset of lesions undergoing dysplastic and malignant transformation. It is therefore suggested that therapeutic interventions aimed at restoring vascular homeostasis in non-dysplastic disease, combined with close monitoring of *VEGF*-driven neovascularization in dysplastic lesions, may enhance tissue perfusion and limit both ischemic fibrosis and malignant progression.

#### 1.2.2. b*FGF*: Emerging Role in Fibrosis and Malignant Transformation

*FGF*s are recognized as a family of signaling molecules that play a key role in angiogenesis, wound healing, and tissue remodeling [62,63,116]. Although the role of *FGF*s in OSMF has been less extensively investigated than that of *VEGF*, it has been suggested by recent research studies that *FGF* signaling may contribute significantly to both fibrotic progression and malignant transformation in OSMF [64,117].

This role is further supported by quantitative analysis. In a case–control study using enzyme-linked immunosorbent assay (ELISA), significantly elevated salivary bFGF levels were reported in patients with OSMF compared with healthy controls, indicating early fibrogenic activation. Based on these findings, b*FGF* has been highlighted as a potential non-invasive biomarker for early detection and risk assessment of OSMF [61,65].

It has been suggested through research findings that b*FGF* contributes to both early cellular activation and later fibrotic remodeling in OSMF pathogenesis [118]. At the tissue level, stage-specific effects of b*FGF* have been observed. Through immunolocalization studies, consistent upregulation of b*FGF* expression has been demonstrated within the epithelium and underlying connective tissue of OSMF lesions relative to normal mucosa, along with increased expression of profibrotic cytokines and growth factors [110,119]. This pattern suggests that *FGF* signaling contributes to fibroblast activation, increased collagen production, and continued extracellular matrix accumulation. b*FGF* expression in OSMF changes across different stages of the disease. Higher levels are seen in fibroblasts and blood vessels in early OSMF, reflecting early activation of fibroblasts and endothelial cells. In advanced stages, its expression becomes more prominent in the connective tissue stroma, which corresponds with progressive fibrotic remodeling [81,118].

In addition, recent evidence suggests that b*FGF* has a dual role in the pathobiology of OSMF. Its expression decreases as fibrosis progresses, but increases during malignant transformation, suggesting that b*FGF* may act both as a mediator of early fibrosis and also as a marker of tumorigenic potential [120].

Despite these findings, treatment strategies targeting *FGF* signaling in OSMF remain poorly explored, highlighting an important gap between research and clinical application. A better understanding of stage-specific changes in b*FGF* may help develop novel, targeted, stage-specific treatments to control fibrosis and reduce the risk of malignant transformation in OSMF.

#### 1.2.3. *EGF*: Limited Direct Evidence but Potential Mechanistic Relevance

*EGF*, through its receptor *EGFR*, is an important regulator of epithelial growth. It promotes proliferation, migration, and differentiation of keratinocytes, endothelial cells, and fibroblasts. It is secreted by platelets, macrophages, and acts on nearby epithelial cells through paracrine signaling. In addition to its role in tissue repair, *EGF*, along with other growth factors, enhances collagen deposition and neovascularization, linking epithelial regeneration with stromal remodeling and wound healing [66,67]. In OSMF, significant upregulation of *EGF*/*TGF-α–EGFR* signaling has been reported in the epithelium compared with normal mucosa, suggesting that this pathway is involved in epithelial dysregulation and may be linked to malignant transformation rather than fibrosis alone [68,69]. It has been shown that areca nut-induced oxidative stress triggers sustained *EGF* release, creating a self-perpetuating loop through which inflammatory and profibrotic pathways are continuously activated. As a result, chronic epithelial stimulation, disrupted tissue repair, and progressive collagen build-up are maintained, all of which contribute to OSMF pathogenesis [70].

*EGF–EGFR* signaling has also been identified as a key molecular link between oral fibrosis and oral cancer. In the presence of an upregulated TGF-β fibrotic microenvironment, malignant progression has been found to be driven by persistent *EGF* signaling, making *EGF*/*EGFR* a clinically relevant therapeutic target [121]. Since *EGF* and *TGF-β* signaling pathways are functionally connected, targeting both axes together has been proposed as a more effective mechanism-based approach than single pathway interventions for controlling fibrosis and reducing malignant transformation risk in OSMF.

#### 1.2.4. Integration: Angiogenic–Fibrotic Interaction

**Figure 1:**
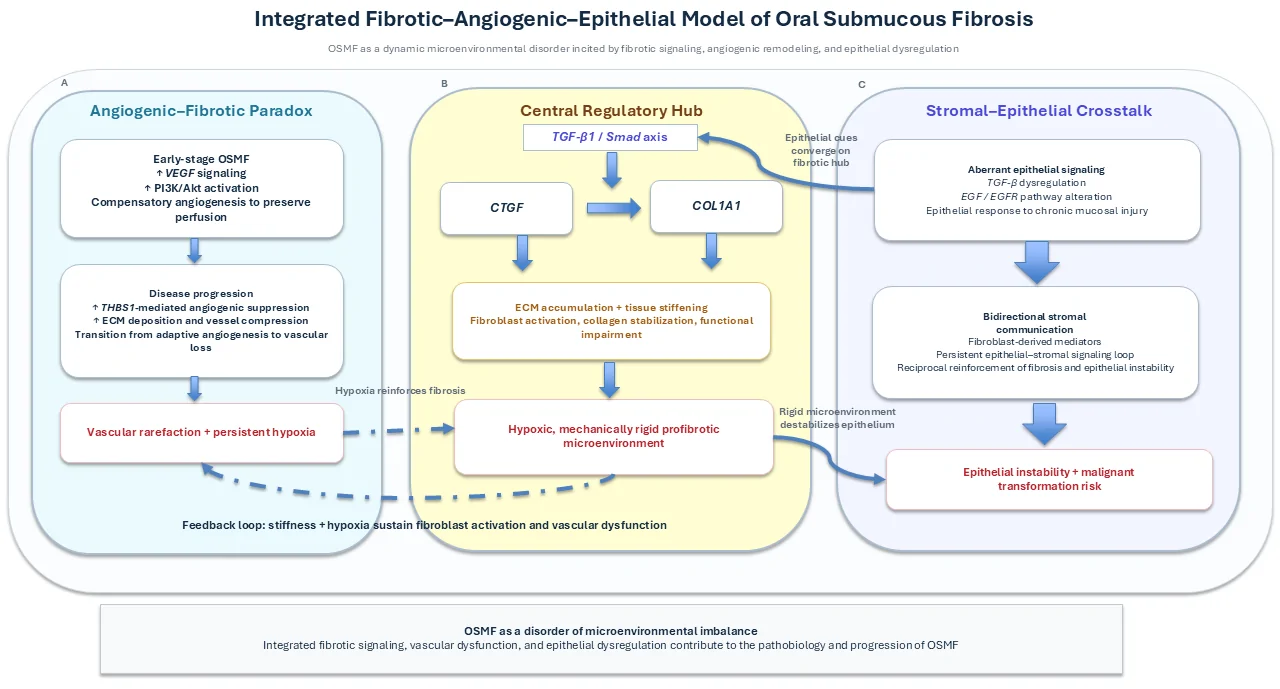
Integrated fibrotic–angiogenic–epithelial model of oral submucous fibrosis (OSMF): (**A**) angiogenic-fibrotic paradox, showing early compensatory angiogenesis followed by THBS1-driven vascular loss and persistent hypoxia; (**B**) central regulatory hub, showing *TGF-β1*/*Smad*-driven *CTGF*/*COL1A1* upregulation leading to ECM accumulation and tissue stiff-ening; (**C**) stromal-epithelial crosstalk, showing aberrant epithelial signaling reinforcing fibrosis and increasing malignant transformation risk. The feedback loop shows hypoxia and stiffness jointly sustaining fibroblast activation and vascular dysfunction.

The interaction between angiogenic factors (*VEGF*, *FGF*, *EGF*) and fibrotic mediators (*TGF-β1*, *CTGF*, *COL1A1*) forms a dynamic network during OSMF progression. In early stages, increased angiogenic signaling (notably *VEGF*/PI3K-Akt) helps maintain tissue perfusion as an adaptive response. As *TGF-β1*-driven fibrosis progresses, anti-angiogenic factors such as *THBS1* dominate, resulting in vascular loss, hypoxia, and further fibrotic signaling through HIF-1α, with *FGF* supporting fibroblast activity and increases malignant risk. This transition explains the blanched, stiff, avascular mucosa seen in advanced OSMF and highlights the importance of effective therapeutic strategies that address both vascular dysfunction and fibrosis together, rather than targeting either process alone. The integrated fibrotic–angiogenic–epithelial interactions described above are summarized in the schematic model shown in Figure 1.

## 2. Discussion

### 2.1. Integrated Fibrotic–Angiogenic Pathobiology of Oral Submucous Fibrosis

Collectively, OSMF can be understood as a complex condition where different processes, such as fibrotic signaling, angiogenic remodeling, and epithelial dysregulation, interact in a coordinated manner rather than reflecting isolated molecular perturbations. Within this integrated framework, the *TGF-β1*/Smad pathway functions as a central regulator coordinating these events [24,35,45,53,76,79]. *CTGF* and *COL1A1*, its downstream mediators, contribute to progressive extracellular matrix accumulation that leads to tissue stiffening and functional limitation [56,74,83,84,85,87,90,95,96,97,98,122]. Notably, angiogenic and epithelial responses evolves in parallel with fibrotic remodeling, collectively shaping a progressively pathological microenvironment.

The angiogenic–fibrotic paradox described in Section 1.2.1 is not simply a descriptive curiosity; it has direct implications for staging and treatment selection. Because fibrotic, angiogenic, and epithelial signaling operate as one coordinated network rather than as separable pathways, a biomarker or intervention validated against a single axis, *VEGF* alone, or *TGF-β1* alone captures only part of the disease state at any given stage [59,60,112,113,114]. A biomarker or therapy validated in early, angiogenically active disease is therefore unlikely to remain useful once the tissue shifts to a fibrosis-dominated, vascularly rarefied state, and the reverse is also true: late-stage biomarkers are unlikely to apply early. Stage-specific interpretation is therefore essential for valid biomarker use and treatment selection in OSMF, rather than an optional refinement.

The growing understanding of how the stroma and epithelium interact during OSMF progression is also important. Aberrant *TGF-β* signaling, along with dysregulated *EGF*/*EGFR* pathways and factors released from fibroblasts, establishes a sustained communication between the epithelial layer and the underlying stromal tissue [68,69].

This two-way communication disturbs the epithelial homeostasis along with perpetuating fibrogenesis, potentially linking fibrosis to malignant transformation risk [54,70] (Figure 1). From this perspective, OSMF should be seen as a disorder caused by an imbalance in the tissue microenvironment, rather than a pathological condition caused solely by fibroblast activation [70]. This broader view brings together molecular, histopathological, and clinical features into a more unified framework. It provides better insight into how the disease progresses and also helps in identifying possible targets for more effective treatment strategies.

### 2.2. Why Current Therapies Fail

Despite decades of research, therapeutic outcomes in OSMF remain largely suboptimal. Most available treatments mainly offer symptomatic relief rather than significantly altering disease progression. This shows a gap between current therapeutic paradigms and the complex and evolving nature of the disease. Clinically, most approaches focus on controlling inflammation or alleviating symptoms [45,50,123,124,125,126,127,128]. However, fibrosis in OSMF becomes self-sustaining through *TGF-β*-driven autocrine signaling, *CTGF* amplification, and ECM-mediated mechanotransduction. Once established, this profibrotic network operates independently of the initial inflammatory trigger, thereby limiting the long-term efficacy of conventional anti-inflammatory therapies.

A further limitation is due to the lack of stage-specific therapeutic stratification [45,60,77,82,126]. Evidence indicates that early OSMF is characterized by active and potentially reversible, angiogenic and cellular responses [57,59,109], whereas advanced disease is dominated by vascular rarefaction, matrix stiffening, and irreversible structural remodeling [59,60,109,112,114] (Figure 1). Whether this later-stage remodeling is truly irreversible in OSMF, as in other fibrotic diseases, has not been directly confirmed in the absence of longitudinal human data (Section 2.5); interventions effective during early adaptive phases are therefore plausible, though not yet confirmed, to be less effective in later stages. In parallel, most experimental therapies focus on single molecular target approach [40,45,51,52,53,58,73,74], which fails to capture the integrated nature of OSF pathobiology, where fibrotic, angiogenic, and epithelial signaling networks operate in an interconnected manner. Although these strategies can affect specific molecular pathways, they usually do not restore the overall balance of the tissue microenvironment. Because of this, there is a persistent gap between promising and encouraging preclinical data and its limited clinical translation.

Therefore, these challenges highlight the need for treatment approaches that are adapted to different stages of the disease and target multiple pathways at the same time. Such strategies would be more suited to the evolving and interconnected mechanisms involved in the progression of OSMF.

### 2.3. Implications for Malignant Transformation

The integrated framework also helps explain how OSMF can progress to malignant transformation. Progressive ECM stiffening, chronic hypoxia, and continuous epithelial stress together create an environment that supports tumor development. Increased matrix rigidity disrupts mechanotransduction signaling, promoting epithelial proliferation and resistance to apoptosis, while hypovascularity exacerbates oxidative stress and genomic instability, thereby facilitating malignant progression [34,36,37,47,60,76,115].

In this context, the concept of field cancerization becomes important. A biologically altered mucosal environment is created by OSMF, where tissues are continuously exposed to profibrotic cytokines and dysregulated growth factor signaling [54,129,130]. Concurrent alterations in *EGF* and *FGF* pathways further enhance epithelial plasticity and promote epithelial–mesenchymal transition (EMT), functionally linking fibrotic remodeling to increased oncogenic susceptibility [64,70,81,118,120,121] (Figure 1). From this perspective, malignant transformation in OSMF is a gradual process caused by ongoing interactions between the stroma and epithelium, rather than an isolated pathological event.

### 2.4. Translational and Therapeutic Opportunities

Viewing OSMF as an integrated disorder of the tissue microenvironment highlights several opportunities for therapeutic interventions. Treatments that concurrently reduce profibrotic signaling while restoring vascular homeostasis are likely to be more effective than conventional treatments that target only a single pathway. Within this paradigm, using biomarkers to guide stage-specific intervention offers a promising strategy. Molecular indicators such as *VEGF* signaling patterns, *CTGF* expression, and salivary *TGF-β* isoforms may help patients’ stratification and the identification of the optimal timing for intervention. This may allow early targeting of active fibrotic pathways in initial stages, while, in advanced disease, focus can shift to matrix remodeling along with improving vascular function.

Multiple interconnected pathways can also be simultaneously targeted through emerging modalities such as non-coding RNA-based therapies, extracellular vesicle mediated delivery systems, and tissue engineering techniques like scaffolds loaded with bioactive compounds [78,127,128,131,132]. These approaches are well aligned with the integrated disease model described in this review and show strong potential for improving clinical outcomes in OSMF.

Drug repurposing offers a further, more immediately translatable route. Metformin, a first-line antidiabetic agent with established antifibrotic activity in other organs, has recently generated OSMF-specific evidence: in fibrotic buccal mucosal fibroblasts, metformin suppressed myofibroblast activity via downregulation of the long non-coding RNA MALAT1 [133]. A systematic review of metformin’s antifibrotic effects across preclinical and clinical fibrosis literature similarly concluded that the existing evidence is sufficient to justify dedicated OSMF trials [134]. Because metformin is inexpensive, has a well-characterized long-term safety profile, and targets fibrotic signaling directly rather than only symptoms, it illustrates the kind of repurposed, mechanism-targeted therapy that this integrated framework favors over single-target or purely symptomatic treatments.

### 2.5. Limitations of Current Evidence

Despite progress in understanding the molecular basis of OSMF, some important limitations still remain. Much of the current evidence comes from in vitro experiments or animal studies, which do not fully represent the biological complexity and variation seen in human disease.

The lack of long-term human studies tracking molecular changes across disease stages makes it difficult to establish clear cause-and-effect relationships [122]. Without patient data from different stages of OSMF, it is unclear whether specific molecular changes drive disease progression or simply accompany it, making it harder to identify reliable biomarkers and develop targeted treatments [23,122].

Similar challenges are also seen in the development of new therapies. Although tissue engineering approaches have gained a lot of attention, their application in OSMF is still largely limited to preclinical settings, with insufficient clinical evidence to confirm their safety, effectiveness, and long-term efficacy [75,135,136].

Furthermore, practical challenges are encountered, such as difficulty in achieving controlled and sustained drug release, variable host responses, and issues related to large-scale production, cost, and regulatory approval [127,131]. These factors continue to limit their wider use in clinical practice. These limitations need to be addressed to translate our understanding of disease mechanisms into effective, patient-focused therapeutic strategies.

### 2.6. Future Directions

Future research should be directed towards stage-specific and biologically stratified clinical approaches rather than uniform treatment strategies. Strategies should be designed to account for the dynamic nature of OSMF. Greater effectiveness is likely to be achieved by therapies that target both fibrotic signaling and vascular dysfunction simultaneously [77]. This becomes particularly important when combined with early identification of high-risk molecular markers, which may help prevent progression to oral squamous cell carcinoma (OSCC) [45,77,82,126,134].

Comprehensive long-term studies that integrate molecular profiling with clinical outcomes are very important. These studies can help us better understand how the disease progresses and identify the optimal time points for effective treatment. The development of reliable salivary and tissue-based biomarkers is equally important for monitoring fibrotic activity, assessing malignant potential, and supporting a more precise and personalized approach to managing OSMF [23,33].

## 3. Conclusions

OSMF is no longer seen as just a simple pathological condition of collagen deposition. Instead, it is better understood as a dynamic and progressive disease caused by coordinated dysregulation in fibrotic, angiogenic, and epithelial signaling pathways. In this review, current evidence on the pathobiology of OSMF has been gathered. It is shown that continuous activation of the *TGF-β1*/Smad, *CTGF* and *COL1A1* pathway plays a central role in fibrogenesis in OSMF. Through this pathway, myofibroblasts are activated, leading to excessive extracellular matrix accumulation and progressive tissue stiffening. Importantly, these profibrotic processes do not occur alone but are closely linked with angiogenic dysfunctions that vary with disease stage. In early stages, compensatory increase in *VEGF* activity is seen, but in later stages, this response becomes ineffective due to dominant anti-angiogenic and hypoxia-driven mechanisms.

The emerging concept of the fibrotic–angiogenic paradox helps explain key clinical and histopathological features of OSMF, including mucosal blanching, reduced blood supply, restricted mouth opening, and an increased susceptibility to malignant transformation. Growth factors, such as *VEGF*, *FGF*, and *EGF*, are known for helping in tissue repair, but in OSMF, their roles change within a *TGF-β*-dominated microenvironment. These factors contribute to vascular rarefaction, epithelial dysregulation, and changes in the stroma that increase the risk of malignant transformation. These findings explain why current treatment approaches, which are mostly focused on symptom relief without targeting specific disease mechanisms, have not been successful in stopping or reversing the disease progression.

From a translational perspective, this review emphasizes the need to move beyond targeting single pathways. Instead, it recommends integrated and stage-specific treatment strategies that can simultaneously reduce fibrotic signaling, restore vascular homeostasis, and stabilize epithelial integrity. Targeting shared molecular nodes in the fibrotic and angiogenic pathways, combined with using biomarker-guided patient stratification, provides a logical approach for improving disease outcomes and lowering the risk of malignant transformation in OSMF.

In conclusion, understanding OSMF as a complex disorder involving fibrosis, angiogenesis, and epithelial plasticity provides a strong basis for future research and therapeutic innovation. Advancing this integrated approach into well-designed clinical studies will be important for improving OSMF management. This may help shift treatment from palliative symptom control to approaches that can reverse fibrosis and reduce the risk of malignant transformation.

## Figures and Tables

**Table 1:** Integrated fibrotic and angiogenic signaling in oral submucous fibrosis.

Molecular Axis	Core Role in OSMF Pathogenesis	Disease Stage	Therapeutic Relevance	References
*TGF-β1*/*Smad*	Sustained fibroblast activation, myofibroblast differentiation	All stages	Central antifibrotic target	[34,35,36,37,40,45,46,47,48,49,50,51,52,53]
*CTGF* (*CCN2*)	Amplifies TGF-β1 signaling to enhance collagen deposition and ECM accumulation.	Progressive	Downstream blockade may reduce fibrosis	[45,48,49,50,51,54]
*COL1A1*	Matrix stiffening and mucosal rigidity	Advanced	Targeting ECM remodeling	[30,37,40,52,55,56]
*VEGF*/*PI3K–Akt*	Early compensatory angiogenesis	Early	Vascular restoration strategies	[47,57,58,59]
*THBS1–CD36*	Angiogenic inhibition and vascular rarefaction	Advanced	Anti-angiostatic targeting	[30,59,60]
*FGF* signaling	Fibroblast proliferation and malignant risk	Stage-dependent	Biomarker and adjunct target	[61,62,63,64,65]
*EGF*/*EGFR*	Epithelial dysregulation and malignant transformation risk	Late	Dual antifibrotic–anticancer targeting	[66,67,68,69,70,71]

**Table 2:** Descriptive summary of the studies.

Study (Reference)	Study Design	Experimental Model	Key Biomarkers/ Molecular Targets	Outcome Measures	Therapeutic Agent/ Intervention
Srinivasan & Jewell, 2001 [69]	Clinical cross-sectional observational study	Human tissue biopsies: oral leukoplakia, oral submucous fibrosis (OSMF), and normal oral mucosa (IHC)	*TGF-α*, *EGFR*	*TGF-α* and *EGFR* expression	None
Sobral et al., 2011 [48]	In vitro	Gingival fibroblasts	*TGF-β1*, *CTGF*, α-SMA, Collagen I, Smad2	Fibroblast–myofibroblast transition, collagen expression	Smad7 overexpression
Khan et al., 2011 [36]	Clinical cross-sectional observational study of immuno-histochemistry (IHC)	Human OSMF biopsy sample IHC	*TGF-β1*, *CTGF*, *THBS1*, BMP7, p-Smad2	ECM accumulation, fibrosis severity	None (mechanistic study)
Khan et al., 2012 [37]	In vitro experimental study	Epithelial cells & gingival fibroblasts exposed to areca nut	*TGF-β* pathway genes, α-SMA and γ-SMA, collagen isoforms	Fibroblast activation, contractility	Pathway identification (no drug)
Chang et al., 2013 [50]	In vitro experimental study	Human buccal fibroblasts	*CTGF*, collagen genes, JNK, p38 MAPK	Myofibroblast differentiation	EGCG
Maha et al., 2013 [49]	In vitro experimental study	Gingival fibroblasts	*CTGF*, GSK-3β, PI3K, α-SMA and collagen I expression	Fibrotic gene expression	GSK-3β inhibition
Kamath et al., 2015 [34]	Clinical cross-sectional observational study	OSMF tissues compared with controls (IHC)	*TGF-β1*, *TGF-β2*	Expression correlation with disease stage	None
Meka et al., 2015 [72]	Retrospective immunohistochemical study	Human OSMF, oral leukoplakia, and normal oral mucosa	*EGFR*	*EGFR* expression correlated with malignant risk	None
Pant et al., 2015 [32]	In vitro and in vivo experimental study	Areca nut-treated oral epithelial cells and fibroblasts	*TGF-β*, Smad, epithelial–mesenchymal markers	Epithelial-fibroblast *TGF-β* crosstalk driving fibrotic remodeling	None
Chang et al., 2017 [45]	In vitro experimental study	Fibroblasts (thrombin stimulation)	*TGF-β1*, *CTGF*, Smad3, integrins *αvβ1*, *αvβ3*, and *αvβ5*	Fibroblast activation	EGCG
Singh et al., 2019 [24]	Clinical cross-sectional biomarker correlation study	OSMF patient serum & tissue	α-SMA, *TGF-β1*	Correlation with clinical severity	None
Rai et al., 2020 [35]	Clinical cross-sectional tissue expression study	OSMF biopsies	*TGF-β* isoforms, *TGF-β* receptors	histopathological grading correlation	None
Yu et al., 2021 [55]	In vitro experimental study (lncRNA–miRNA functional analysis)	OSMF fibroblasts	H19, miR-29b, *COL1A1*	Collagen regulation	ncRNA modulation
Shah et al., 2021 [54]	Clinical cross-sectional observational study	OSMF & OSCC tissues (IHC)	*CTGF*	Stage progression correlation	None
Boreak, 2021 [73]	In silico	Molecular docking	*TGF-β1*	Binding affinity prediction	Silmitasertib
Deng et al., 2022 [74]	In vitro experimental study	Gingival fibroblasts	NOX4, *TGF-β1*, *CTGF*	Suppression of *TGF-β1*–CCN2 fibrotic signaling.	Curcumin
Peng et al., 2022 [58]	In silico	Molecular docking	*VEGFA*, ALB in OSMF	Target prediction	Curcuma compounds
Adhikari et al., 2022 [75]	Randomized double-blind controlled trial	Human OSF patients	None (clinical trial)	Mouth opening, tongue protrusion, cheek flexibility, burning sensation	Curcumin + intralesional dexamethasone/hyaluronidase
Chou et al., 2023 [76]	Combined clinical cross-sectional and in vitro experimental study	OSMF tissues and fBMFs	miR-424, *TGF-β*/Smad, *COL1A1*, α-SMA	Contractility, collagen deposition and Migration	None
Bansal et al., 2023 [33]	Retrospective immunohistochemical study	Human NOM, OSF (stage 1–4), and OSCC tissue	*TGF-β1*, *TGF-β3*	*TGF-β* isoform expression across OSF stages and malignant transformation	None
Mehta et al., 2023 [40]	In vivo + in vitro	OSMF rat model + fibroblasts	*TGF-β1*, Collagen I	Myofibroblast activity and mouth-opening improvement	EGCG hydrogel
Jiang et al., 2023 [57]	Clinical cross-sectional observational study (tissue analysis)	Normal tissue and OSMF tissues at different stages	*VEGF*, PI3K/Akt	Stage-dependent angiogenesis	None
Chang et al., 2023 [51]	In vivo experimental study	Animal model (ANE treated mouse skin)	*CTGF*, α-SMA	Fibroblast activation and collagen deposition	Simvastatin
Umamaheswari T, Jency Evanjelin et al., 2023 [77]	In vitro	OSMF-derived fBMFs	*TGF-β*, SMAD2, *COL1A1* and α-SMA	Fibrogenic activity	MPE extract
Lee et al., 2023 [52]	In vitro	OSMF-derived fibroblasts	Linc-ROR, *TGF-β1*, α-SMA and *COL1A1*	Myofibroblast differentiation, migration, and contractility	α-Mangostin
Wang et al., 2023 [46]	Clinical observational study	OSMF tissue samples	*TGF-β1*, SASP factors	Epithelial senescence	Target hypothesis
Wang et al., 2023 [78]	In vivo experimental therapeutic study	ADSC-EV-treated models	*TGF-β1*, Smad3, miR-760-3p, MMPs and TIMPs	Enhanced antifibrotic efficacy	ADSC-derived EVs
Rai et al., 2023 [79]	Clinical cross-sectional biomarker study	Saliva samples	*TGF-β* isoforms	Biomarker levels reflect disease activity	None
Yang et al., 2024 [60]	In vivo + in vitro	OSMF models	*THBS1*, CD36	Angiogenesis suppression	*THBS1* inhibition
Roghay et al., 2025 [61]	Clinical cross-sectional biomarker study	Salivary analysis in OSMF patients vs controls	b*FGF*	Diagnostic association	None
Mahajan et al., 2025 [80]	Non-randomized pilot clinical trial	Human OSMF patients (Stage 2)	*TGF-β* pathway (antifibrotic target)	Mouth opening, burning sensation, mucosal flexibility	Oral Pirfenidone
Kaur et al., 2025 [81]	Retrospective immunohistochemical study	Human OSF tissue across histopathological grades	Basic fibroblast growth factor (b*FGF*)	b*FGF* expression across disease stages	None
Narvekar et al., 2025 [82]	In vitro experimental study	OSMF-derived human fibroblasts	*TGF-β1*	Fibroblast viability and ECM contraction	Swertia purpurascens extract

## Data Availability

The raw data supporting the conclusions of this article will be made available by the authors on request.

## Abbreviations

.

ECM	Extracellular matrix
OSMF	Oral submucous fibrosis
OSCC	Oral squamous cell carcinoma
WDSCC	Well-differentiated squamous cell carcinoma
*TGF-β1*	Transforming growth factor-β1
*TGF-β2*	Transforming growth factor-β2
*TGF-β3*	Transforming growth factor-β3
*CTGF*	Connective tissue growth factor
*COL1A1*	Collagen type I alpha 1 chain
*VEGF*	Vascular endothelial growth factor
*FGF*	Fibroblast growth factor
b*FGF*	Basic fibroblast growth factor
*EGF*	Epidermal growth factor
*EGFR*	Epidermal growth factor receptor
PI3K/Akt	Phosphoinositide 3-kinase/protein kinase B (Akt) signaling pathway
*THBS1*	Thrombospondin-1
CD36	Cluster of differentiation 36
EMT	Epithelial–mesenchymal transition
SASP	Senescence-associated secretory phenotype
COMP	Cartilage oligomeric matrix protein
*MMP*	Matrix metalloproteinase
*TIMP*	Tissue inhibitor of metalloproteinases
HIF-1α	Hypoxia-inducible factor-1 alpha
α-SMA	Alpha-smooth muscle actin
ROS	Reactive oxygen species
lncRNA	Long non-coding RNA
miRNA	MicroRNA
EVs	Extracellular vesicles
BMP7	Bone morphogenetic protein 7
PAI-1	Plasminogen activator inhibitor-1
JNK	c-Jun N-terminal kinase
GSK-3β	Glycogen synthase kinase-3 beta
CD105	Endoglin (cluster of differentiation 105)
CD34	Cluster of differentiation 34
Ki67	Ki-67 proliferation marker
hTERT	Human telomerase reverse transcriptase
IHC	Immunohistochemistry
qPCR	Quantitative polymerase chain reaction
ELISA	Enzyme-linked immunosorbent assay
ANE	Areca nut extract
HaCaT	Human keratinocyte cell line
